# Indirect Path from Cyberbullying to Suicide Attempts: Hopelessness as a Central Bridge in a Risk Behavior Network

**DOI:** 10.3390/bs16071065

**Published:** 2026-06-26

**Authors:** Jiaxin Hu, Lijun Ma, Xu He

**Affiliations:** 1 Department of Psychology, Guangzhou University of Chinese Medicine, Guangzhou 510006, China; 2 School of Psychology, South China Normal University, Guangzhou 510631, China

**Keywords:** cyberbullying, network analysis, hopelessness, suicidal behavior, youth

## Abstract

Despite growing concern about cyberbullying as a contributor to the adolescent mental health crisis, its position within the broader network of co-occurring risks remains theoretically unresolved. Guided by the Three-Step Theory of suicide, the current study conceptualized cyberbullying as a distal contextual risk that influences suicidality indirectly through hopelessness. An Ising model network was estimated in a nationally representative sample of 9621 U.S. high school students from the 2023 Youth Risk Behavior Survey, including cyberbullying victimization and 13 other risk behaviors. The results showed that hopelessness was the most central node (Strength z = 1.89) and the strongest bridge (Bridge Strength z = 2.35), linking mental health to other domains. The shortest path from cyberbullying to suicide attempts was direct (path length = 2.37), though the indirect pathway through hopelessness and suicidal ideation was marginally longer (2.48), and removing hopelessness reduced cyberbullying’s bridge strength from 3.00 to 2.39 (Δ = −0.61). Network comparison tests revealed no significant sex differences in global strength or structure, and bootstrap analyses confirmed excellent stability. These findings position hopelessness as a central bridging node in the adolescent risk network.

## 1. Introduction

Adolescent mental health has deteriorated markedly over the past decade, with rising rates of depression, anxiety, and suicidality among high school students (Verlenden et al., 2024). The COVID-19 pandemic exacerbated these trends, with the U.S. Surgeon General issuing an advisory declaring a youth mental health crisis (Office of the Surgeon General, 2021). Among the risk factors implicated in this crisis, cyberbullying has emerged as a particularly salient concern, given the pervasive role of digital technology in adolescents’ daily lives (Odgers & Jensen, 2020). Recent studies have shown the co-occurrence of bullying victimization with other forms of violence and adverse mental health outcomes (Weinreich et al., 2023; Ghardallou et al., 2024) and the association between frequent social media use, bullying victimization, and elevated suicide risk (Young et al., 2024).

Cyberbullying is defined as repeated, intentional aggression through digital media. Unlike face-to-face victimization, it can reach victims in safe spaces like home, creating a sense of inescapability (Akdeniz & Doğan, 2024). Critically, cyberbullying rarely occurs in isolation. It is commonly accompanied by traditional bullying, depression, substance use, aggression, and school difficulties, forming an interconnected risk cluster (Bansal et al., 2024). Consistent with these co-occurrence patterns, meta-analytic evidence confirms strong associations with depression, anxiety, suicidal behavior, self-harm, and suicidal ideation (Brailovskaia et al., 2018; Eyuboglu et al., 2021). Longitudinal studies further demonstrate that cybervictimization prospectively predicts depression and suicidal ideation (Perret et al., 2020). Recent findings similarly confirm the co-occurrence of cyberbullying victimization and depressive symptoms in youth (Teixeira et al., 2025). Notably, sleep disturbance, self-esteem, and loneliness have been identified as key bridge symptoms that help explain how cybervictimization connects to depression and anxiety (Xiao et al., 2025).

Traditional variable-centered approaches impose directional assumptions that may not reflect how symptoms actually interact (Borsboom et al., 2021). Network analysis offers a fundamentally different perspective by mapping each symptom as an interconnected element and estimating how they relate to one another after controlling for all others, thereby revealing the architecture of comorbidity (Borsboom et al., 2021). Estimation typically applies penalized regression techniques to produce a sparse, interpretable map of these relationships (Marsman et al., 2018; Van Borkulo et al., 2023). A key strength is identifying symptoms that link otherwise distinct clusters, quantified by bridge centrality, which helps pinpoint where intervention might most effectively disrupt co-occurring problems (Jones et al., 2021). This approach has been successfully applied to depression, anxiety, adolescent suicidality, and co-occurring victimization patterns (De Vries et al., 2022; Fonseca-Pedrero et al., 2022; F. Li et al., 2024).

Despite the growing application of network analysis to psychopathology, the theoretical architecture linking cyberbullying to its most commonly co-occurring risk behaviors has not been clearly specified. In particular, existing studies have not embedded their network findings within suicide theories, which distinguish between the development of suicidal ideation and the progression to suicidal behavior (Klonsky et al., 2018). The Three-Step Theory (Klonsky et al., 2021) positions hopelessness as the central mechanism through which distal stressors may influence suicidal outcomes. In parallel, the buffering hypothesis of resilience proposes that protective factors (e.g., self-efficacy, subjective happiness) can attenuate the impact of risk factors on suicidal potential (Johnson et al., 2011). A recent longitudinal network analysis by Y. Li and Kwok (2023) integrated these frameworks and demonstrated that among early adolescents, hopelessness prospectively predicted suicidal ideation and acts, while self-efficacy and subjective happiness buffered these risk pathways.

Drawing on this integrated framework, a parsimonious model of adolescent risk networks can be specified, in which cyberbullying operates as a distal contextual stressor whose influence on suicidal ideation and behavior is largely indirect, transmitted through hopelessness as a transdiagnostic bridge state. Xie et al. (2023) and Fonseca-Pedrero et al. (2022) provided initial evidence identifying bridge symptoms, but because they used clinical or convenience samples and narrow variable sets, they do not clarify how cyberbullying is embedded in the wider risk network of the general adolescent population. It remains theoretically unresolved whether cyberbullying operates as a central hub that activates multiple problem domains, functions as a bridge connecting otherwise distinct clusters of psychopathology, or primarily operates as a distal stressor whose influence is transmitted through proximal intra-individual states. Without a generalizable network model that specifies the topological position of cybervictimization within adolescent mental health and that is grounded in formal suicide theories, the field lacks the theoretical grounding needed to derive network-informed intervention targets that can be applied beyond clinical subgroups.

The present study applied network analysis to a nationally representative sample of U.S. high school students, estimating the conditional dependence structure of cyberbullying victimization and 13 co-occurring risk behaviors including traditional bullying, substance use, depressive symptoms, and suicidality. Informed by the Three-Step Theory, we conceptualized cyberbullying and traditional bullying as distal contextual risks and hopelessness and suicidal ideation as proximal intra-individual risk states. To clarify the theoretically unresolved position of cyberbullying within adolescent risk networks, we tested whether it functions as a bridge linking externalizing and internalizing clusters and, critically, whether its connection to suicidal ideation is direct or indirect. We hypothesized that (a) hopelessness would show the highest bridge centrality across domains; (b) cyberbullying would show strong edges with traditional bullying but only weak direct connections to suicidal ideation; and (c) the shortest path from cyberbullying to suicide attempts would traverse hopelessness and suicidal ideation, indicating indirect transmission.

## 2. Materials and Methods

### 2.1. Participants

Data for this study were drawn from the 2023 national Youth Risk Behavior Survey (YRBS), a school-based, cross-sectional survey conducted by the Centers for Disease Control and Prevention (CDC) that monitors health-risk behaviors among U.S. high school students in grades 9–12. The YRBS employs a three-stage cluster sampling design to obtain a nationally representative sample of public and private school students. After applying listwise deletion for any missing values on the 14 analysis variables, the final analytic sample consisted of N = 9621 adolescents, including 4883 males (50.9%) and 4709 females (49.1%). The 2023 YRBS data and documentation are publicly available from the CDC at https://www.cdc.gov/yrbs/data/index.html (accessed on 8 March 2026).

### 2.2. Measures

Variables from the 2023 YRBS questionnaire were converted into dichotomous (0/1) indicators by the curators of a publicly available compiled dataset (https://github.com/ccani007/dissertationData, accessed on 8 March 2026) prior to this study, with a value of 1 denoting the presence of the risk behavior or experience and 0 denoting its absence. From these, fourteen variables were selected based on the literature review and theoretical frameworks, spanning four domains: bullying, mental health, substance use, and school environment (see Table 1).

### 2.3. Network Estimation and Analysis

Because all analysis variables are binary, the conditional dependence structure was estimated using an Ising model via L1-penalized logistic regression. The regularization parameter was selected per node by minimizing the Extended Bayesian Information Criterion (EBIC) with γ = 0.25. This penalty level is the default in the IsingFit R package and has been shown to yield consistent model selection for binary networks (Van Borkulo et al., 2014). The Ising model was estimated using the IsingFit package in R, which implements neighborhood selection via L1-penalized logistic regression with EBIC-based tuning. Strength, betweenness, closeness, and expected influence centrality were computed and z-standardized (Robinaugh et al., 2016; Bringmann et al., 2019). Bridge centrality was computed to identify nodes that connect otherwise distinct communities, following the method of Jones et al. (2021). Centrality measures were computed using the qgraph package in R, and bridge centrality was computed using the networktools package in R. The four communities (Bullying, Mental Health, Substance Use, School & Environment) were assigned a priori based on theoretical construct domains rather than recovered from the data; bridge centrality values depend on this assignment. NetworkX version 2.6.3 (Hagberg et al., 2008) in Python version 3.9.7 was used for deterministic visualization and shortest-path computation based on the estimated edge-weight matrix, with distance defined as the inverse of the absolute edge weight (1/|w|, where w denotes the pairwise conditional dependence strength). Case-dropping bootstrap yielded correlation stability coefficients (CS-coefficient), with CS ≥ 0.25 considered adequate and ≥0.50 good (Epskamp & Fried, 2018).

## 3. Results

### 3.1. Network Structure

**Figure 1:**
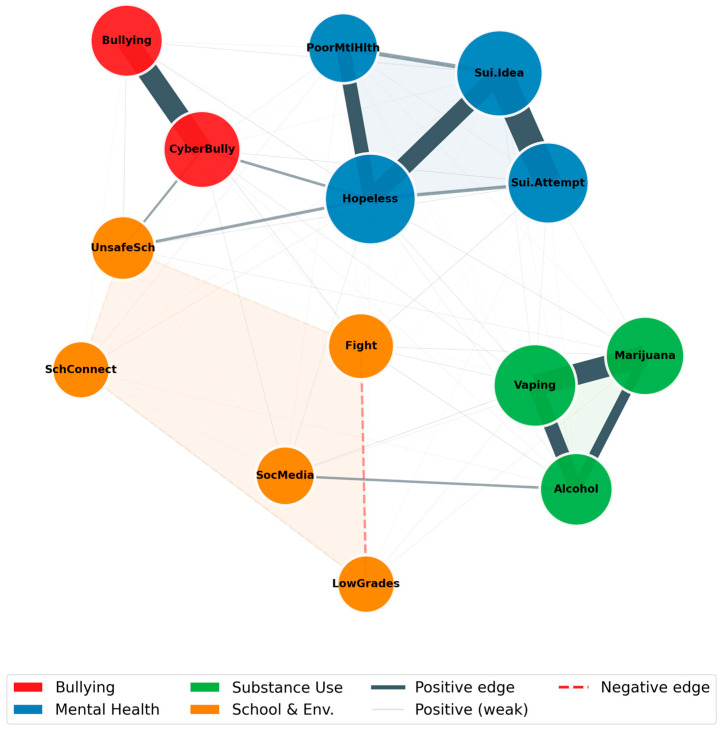
Adolescent Risk Behavior Network. Edge thickness represents absolute weight.

The Ising model estimated a dense network with 71 of 91 possible edges retained (78.0% density; see Figure 1). The three strongest edges in the network were Suicide Ideation–Suicide Attempts (weight = 3.05), Currently Vaping–Currently Using Marijuana (2.40), and CyberBullying–Bullying (2.36). These edges represent the strongest conditional dependencies in the network, controlling for all other variables.

**Figure 2:**
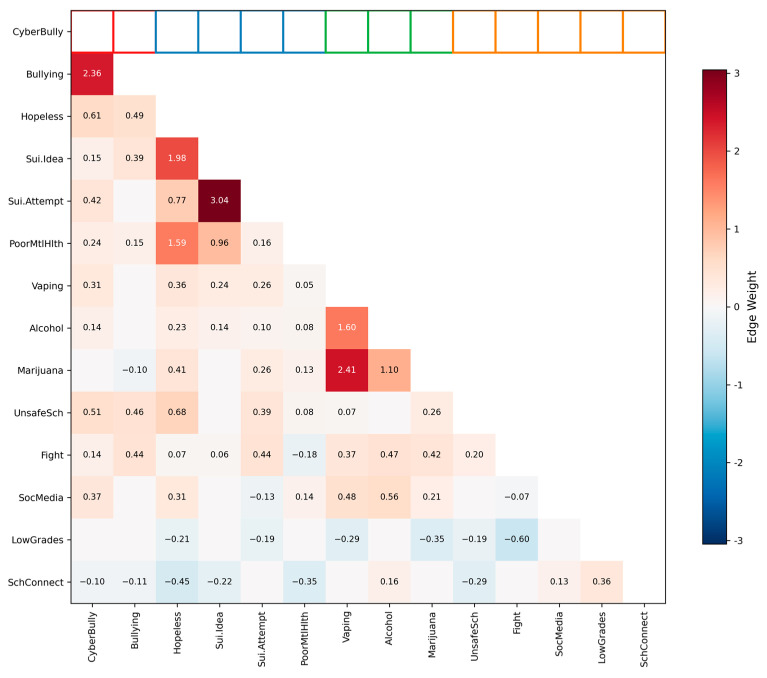
Edge Weight Heatmap.

CyberBullying showed the strongest conditional dependence with traditional bullying (weight = 2.36; see Figure 2). CyberBullying was directly connected to Hopelessness (0.61) and Suicide Ideation (0.15), though the latter edge was very weak. A direct edge from CyberBullying to Suicide Attempts was also retained (0.42). Substance use variables formed a distinct cluster with weaker direct connections to CyberBullying.

### 3.2. Centrality Analysis

Hopelessness had the highest strength centrality (Strength = 8.15, z = 1.89; see Table 2) and expected influence (6.82, z = 1.29). SuicideIdeation ranked second in both strength (Strength = 7.20, z = 1.38) and expected influence (6.76, z = 1.26). CurrentlyVaping ranked third in strength (Strength = 6.45, z = 0.97). CyberBullying ranked sixth in strength centrality (Strength = 5.36, z = 0.37) but fourth in expected influence (5.16, z = 0.63), reflecting its predominantly positive edge weights.

**Figure 3:**
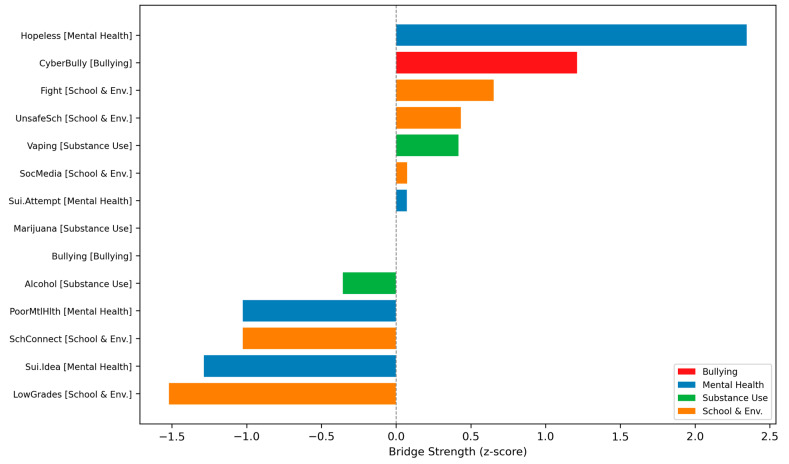
Bridge Centrality by Variable and Community.

Hopelessness was the strongest bridge node (Bridge Strength = 3.81, z = 2.35; see Figure 3). CyberBullying ranked second in bridge strength (Bridge Strength = 3.00, z = 1.22), followed by PhysicalFight (Bridge Strength = 2.60, z = 0.65). CurrentlyVaping was the strongest bridge node in the Substance Use community (Bridge Strength = 2.43, z = 0.42).

The shortest path from CyberBullying to Suicide Attempts was direct (CyberBullying → Suicide Attempts; path length = 2.37), reflecting a retained direct edge after conditioning (0.42). However, the CyberBullying–Suicide Ideation edge was very weak (0.15), whereas the Hopelessness–Suicide Ideation edge was substantially stronger (1.98). The indirect path through Hopelessness and Suicide Ideation (CyberBullying→Hopelessness→Suicide Ideation→Suicide Attempts; path length = 2.48) was longer than the direct path. However, bootstrap confidence intervals (200 resamples) for both path lengths showed substantial overlap: the direct path 95% CI was [1.55, 2.74] and the indirect path 95% CI was [2.14, 3.02]. In 65.5% of bootstrap samples, the indirect path was longer than the direct path, suggesting that the ordering of these paths is unstable under resampling and should not be overinterpreted. Removing Hopelessness from the network reduced CyberBullying’s bridge strength from 3.00 to 2.39 (Δ = −0.61).

### 3.3. Additional Analysis

A sensitivity analysis was conducted to assess the robustness of the EBIC γ = 0.25 network against variations in the penalty tuning parameter. Networks estimated with γ = 0 yielded 75 edges, and γ = 0.5 yielded 64 edges. Edge-weight correlations with the primary γ = 0.25 network were r = 0.999 (γ = 0) and r = 0.998 (γ = 0.5). Centrality rank-order correlations exceeded r = 0.99 for γ = 0 and r = 0.99 for γ = 0.5, indicating that the relative importance of nodes and the network topology were robust to the choice of EBIC tuning parameter. The Network Comparison Test revealed no significant sex differences in global network strength (Male = 30.59, Female = 29.03, difference = 1.56, *p* = 0.422) or network structure (maximum edge difference *p* = 0.344).

To address the concern that the strong SuicideIdeation–SuicideAttempts edge (3.05) reflects item-level redundancy rather than a psychological process, a sensitivity analysis was conducted, removing SuicideIdeation from the network. CyberBullying’s bridge strength remained essentially unchanged (Δ = 0.005), indicating that its bridging role is not an artifact of the suicidality cluster dependency. Hopelessness’s bridge strength increased slightly (Δ = 0.32), suggesting that its bridging role is comparatively more sensitive to the removal of SuicideIdeation, consistent with a shift in the distribution of cross-community connectivity.

**Figure 4:**
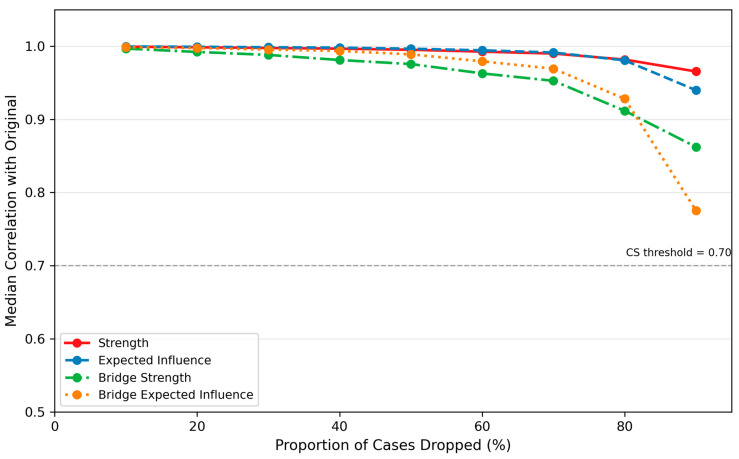
Case-Dropping Bootstrap: Correlation Stability Coefficients for Edges and Centrality.

CS-coefficients (the maximum proportion of cases that can be dropped while maintaining a median correlation ≥0.7 with the original sample; see Figure 4) were uniformly 0.90 for all centrality measures (Strength, Expected Influence, Bridge Strength, and Bridge Expected Influence), indicating excellent stability. Edge-weight bootstraps (500 resamples) showed that 48 of 91 edges (52.7%) had 95% CIs excluding zero, further supporting the reliability of the estimated network structure.

## 4. Discussion

This study applied network analysis to a nationally representative sample of U.S. adolescents to map the conditional dependence structure of cyberbullying and 13 co-occurring risk behaviors. Drawing on the Three-Step Theory and the buffering hypothesis of resilience, we conceptualized cyberbullying as a distal contextual risk whose influence on suicidal behavior should be indirect, operating through hopelessness as the central cognitive–affective bridge state. Three principal findings emerged. First, hopelessness was the most central node in the adolescent risk behavior network and the strongest bridge node linking the mental health domain to bullying, substance use, and school environment domains. Second, the shortest path from cyberbullying to suicide attempts was direct, though bootstrap analyses showed that the direct and indirect paths were comparable in length and unstable under resampling. Third, network structures did not differ significantly by sex. Together, these findings position cyberbullying as one node within a broader risk system, with hopelessness occupying a bridging role.

The finding that hopelessness was the most central node and the strongest bridge aligns with prior network studies of adolescent psychopathology. Specifically, Liu et al. (2021) identified it as the central symptom node in general adolescent samples, and Fonseca-Pedrero et al. (2022) as a key bridge between risk and protective factors for suicidal behavior. In cyberbullying contexts, Xie et al. (2023) found that sad mood, rather than hopelessness, bridged victimization to residual depressive symptoms among clinically stable adolescents, while Wang and Wang (2023) confirmed hopelessness as a direct bridge to suicidal ideation in Chinese community samples, offering cross-cultural convergence. Lu et al. (2025) further showed that bullying and cybervictimization are linked to suicidality through internalizing pathways. The present findings extend this by demonstrating hopelessness as a systemic hub, not merely a context-specific connector, activating robust associations across bullying, mental health, substance use, and school environment.

The strong conditional dependence between cyberbullying and traditional bullying confirms that online and offline victimization co-occur even after controlling for shared variance with other risk behaviors. This is consistent with meta-analytic evidence (Gini et al., 2018) and with the social–ecological model’s emphasis on the overlap between digital and physical peer environments (Patel & Quan-Haase, 2024). CyberBullying’s role as the second strongest bridge node supports the hypothesis that it links the bullying domain to mental health and school environment domains (Borsboom et al., 2021; Jones et al., 2021). However, the weak direct edge between cyberbullying and suicidal ideation relative to the strong hopelessness–suicidal ideation edge indicates that cyberbullying is best understood as a distal contextual risk rather than a proximal cause of suicidal behavior: its influence on suicidality is largely indirect, transmitted through hopelessness.

Contrary to some prior evidence of sex differences in cyberbullying consequences (Barlett & Coyne, 2014), the present study revealed no significant sex difference in global network strength or structure. This finding has several possible explanations. Given the large sample size, this clearly non-significant *p*-value indicates that any potential sex difference is negligible or nonexistent. Sex differences in cyberbullying may be more pronounced at the bivariate level than at the multivariate network level. Moreover, the positive edge between school connectedness and Hopelessness was unexpected and contradicts the social–ecological model’s prediction that school connectedness is protective (Guo et al., 2021). The YRBS item may capture social belonging rather than protective buffering; adolescents who are more emotionally engaged with their school community may also be more vulnerable to distress when that community fails them. It should also be noted that in regularized network models, conditioning on common causes can produce positive edges between variables that are marginally negatively associated, and this reversal may partly explain the unexpected direction of this edge.

Several limitations should be noted, though they do not weaken the main findings. First, the YRBS hopelessness item conflates persistent sadness with the future-oriented hopelessness specified by the 3ST (Klonsky et al., 2021) and functions more like a depression screener. Therefore, the central node likely represents a broader cognitive–affective distress state rather than pure hopelessness. Nevertheless, hopelessness and depressed mood are highly correlated in adolescents (Liu et al., 2021). The core finding that this distress state bridges cyberbullying to suicidality remains interpretable regardless of labeling. Future studies using validated measures are needed to test whether the observed bridging role holds for hopelessness narrowly defined. Second, the cross-sectional design prevents causal inferences. The directional language used in this manuscript is a theory-motivated interpretation rather than empirical evidence of temporal precedence. Nonetheless, the network structure aligns with longitudinal and meta-analytic evidence, which supports the plausibility of hopelessness acting as a bridge symptom. Third, binary coding discards information about severity and frequency. While this conservative approach likely weakens associations, it may also mask heterogeneity in the strength of associations at different severity levels. For example, adolescents experiencing frequent or severe cyberbullying may show stronger connections to mental health outcomes than those experiencing occasional victimization, and this dose–response relationship is not captured by binary indicators. Future studies with ordinal or continuous measures of cyberbullying frequency could examine whether edge weights strengthen at higher severity levels. Future intensive longitudinal studies are needed to examine the real-time link between cyberbullying and suicidal ideation.

These findings have practical implications for school-based mental health. The bridging role of hopelessness suggests that screening for cognitive–affective distress among cyberbullying victims may efficiently identify students at elevated suicidal risk. School professionals could integrate brief hopelessness or sadness items into existing bullying response protocols, particularly for electronically victimized students. Given its high centrality and dense connections to substance use, school disengagement, and suicidality, hopelessness represents a promising transdiagnostic intervention target; interventions addressing hopelessness cognitions may yield cascading benefits across multiple risk domains. Moreover, prevention should move beyond addressing cyberbullying in isolation toward a systemic approach that strengthens school connectedness while directly targeting maladaptive cognitive–affective states, thereby disrupting reinforcing feedback loops that sustain the broader risk network.

## 5. Conclusions

The network analysis reveals that hopelessness, not cyberbullying itself, is the critical transdiagnostic bridge linking peer victimization to suicidality in the adolescent risk behavior network. Cyberbullying is a significant bridge node, but its connection to suicide attempts is primarily bridged through hopelessness, which connects to suicidal ideation and other risk domains. This pattern is consistent with a theoretical model in which distal contextual risks are associated with suicidal outcomes through proximal cognitive–affective states.

## Figures and Tables

**Table 1:** Variable Descriptions, Coding, and Domain Assignments.

Variable	Description	Coding	Domain
CyberBullying	Electronically bullied, past 12 months	0/1	Bullying
Bullying	Bullied on school property, past 12 months	0/1	Bullying
Hopelessness	Felt sad/hopeless ≥2 weeks, past 12 months	0/1	Mental Health
SuicideIdeation	Seriously considered suicide, past 12 months	0/1	Mental Health
SuicideAttempts	Attempted suicide, past 12 months	0/1	Mental Health
NotGoodMentalHealth	Mental health not good ≥1 day, past 30 days	0/1	Mental Health
CurrentlyVaping	Used e-cigarettes, past 30 days	0/1	Substance Use
CurrentlyAlcohol	Drank alcohol, past 30 days	0/1	Substance Use
CurrentlyUsingMarijuana	Used marijuana, past 30 days	0/1	Substance Use
PhysicalFight	In physical fight, past 12 months	0/1	School & Env.
UnsafeAtSchool	Did not go to school due to safety	0/1	School & Env.
SocialMedia	Social media ≥3 h/day	0/1	School & Env.
GradesSchool	Mostly C’s or lower	0/1	School & Env.
SchoolConnectedness	Feel close to people at school	0/1	School & Env.

**Table 2:** Centrality Measures for All Nodes.

Variable	Strength	z	Expected Influence	z	Betweenness	Closeness
Hopelessness	8.15	1.89	6.82	1.29	0.35	0.44
SuicideIdeation	7.20	1.38	6.76	1.26	0.17	0.38
CurrentlyVaping	6.45	0.97	5.88	0.97	0.08	0.34
SuicideAttempts	6.16	0.81	5.52	0.77	0.05	0.37
CurrentlyUsingMarijuana	5.65	0.53	4.74	0.53	0.12	0.35
CyberBullying	5.36	0.37	5.16	0.63	0.04	0.37
CurrentlyAlcohol	4.58	−0.05	4.58	0.40	0.01	0.31
Bullying	4.50	−0.10	4.07	0.04	0.04	0.33
NotGoodMentalHealth	4.12	−0.31	3.04	−0.21	0.0	0.36
PhysicalFight	3.47	−0.66	1.77	−0.72	0.06	0.35
UnsafeAtSchool	3.13	−0.85	2.17	−0.56	0.0	0.32
SocialMedia	2.38	−1.25	1.99	−0.63	0.0	0.28
GradesSchool	2.19	−1.36	−1.48	−2.01	0.01	0.27
SchoolConnectedness	2.18	−1.36	−0.89	−1.78	0.0	0.26

## Data Availability

The data used in this study were obtained from the Youth Risk Behavior Surveillance System (YRBSS), which is a national school-based survey conducted biennially by the Centers for Disease Control and Prevention (CDC). The YRBSS monitors priority health risk behaviors among U.S. high school students, including those related to mental health, substance use, violence, and sexual behaviors. The dataset is publicly available for download from the CDC’s official YRBSS website. The data used in this analysis can be accessed at https://www.cdc.gov/yrbss/data/index.html (accessed on 8 March 2026).
